# Finger Tapping Outperforms the Traditional Scale in Patients With Peripheral Nerve Damage

**DOI:** 10.3389/fphys.2018.01361

**Published:** 2018-10-01

**Authors:** Lingli Zhang, Le Lei, Yilong Zhao, Rong Wang, Yulian Zhu, Zhusheng Yu, Xiaojing Zhang

**Affiliations:** ^1^School of Kinesiology, Shanghai University of Sport, Shanghai, China; ^2^Putuo Hospital, Shanghai University of Traditional Chinese Medicine, Shanghai, China; ^3^Shanghai Fudan University Affiliated Huashan Hospital, Shanghai, China

**Keywords:** peripheral nerve, affected hand, dominant hand, finger tapping, Lind-mark

## Abstract

**Objective:** This study aimed to investigate whether there exist the limits of finger tapping frequency in the peripheral nerve injury detection in upper limb, and the effects of rehabilitation treatment on upper limb with peripheral nerve injury through finger tapping.

**Methods:** Here, 54 patients with peripheral nerve injury in upper limb were selected. We conducted finger tapping frequency test and Lind-mark hand function assessment score on the 54 subjects, and recorded the data 2-week before and after rehabilitation treatment.

**Results:** Finger tapping frequency and Lind-mark hand function assessment score have a high positive correlation regardless of the side of upper limb with peripheral nerve injury before and after the rehabilitation treatment. Finger tapping frequency of the right affected hand after treatment is significantly higher than that of before treatment (male: *P* < 0.05; female: *P* < 0.01), while finger tapping frequency of the left affected hand after treatment shows no significant difference compared to before treatment. Meanwhile, finger tapping frequency of the female subjects' unaffected hand after treatment is significantly higher than before treatment (left: *P* < 0.01; right: *P* < 0.05), however, this was not observed in male subjects. Based on data analysis, there is a high-correlation between finger tapping frequency and Lind-mark score of the patients' affected hand with brachial plexus nerve injury. A trend of the patients' affected hand with radial nerve injury is similar with brachial plexus nerve injury.

**Conclusion:** Compared with Lind-mark score, finger tapping frequency outperformed in the aspect of speed of neural impulse conduction in patients with peripheral nerve damage.

## Introduction

Touch perception in the finger is an indispensable part of fine motor control, which helps contribute to the sense of self, communication, and a clear perception of the world (Tan et al., 2014). Hand functions include percussion, swinging, grasping, pinching, pulling, and pushing, etc. Compared with other neurologically actuated motor tasks, finger tapping has the advantages of the limited inertial and intersegmental interactions helping to reduce bio-mechanical impact on body movement (Liu et al., 2006). The ability and frequency of finger tapping is a key indicator for neuromuscular integrity (Collyer et al., 1994; Cook et al., 2017). Previous studies have applied methods similar to finger tapping to determine handedness of the subjects. It has been previously shown for handedness (Aoki et al., 2016), for some individual differences in skill acquisition (Ackerman and Cianciolo, 2000), and in clinical neurological examinations (Stamatakis et al., 2013).

Loss of even part of the upper limb is a devastating injury (Irwin et al., 2016). As a common disease in the hand surgery department, upper limb peripheral nerve injury is typically caused by productive labor accidents, such as cutting, contusion, and inserting by falling sharp instruments. Obstetric brachial plexus palsy (OBPP) together with medical accidents resulted from intravenous injections are two of the factors which lead to the patients' suffering of upper limb damage or degeneration.

In the present study, we aimed to analyze the correlation between finger tapping and the traditional hand function evaluation scales of the affected hand of patients with upper limb peripheral nerve injury to provide a reference for tapping frequency applications in hand function assessment. We attempted to find out whether finger tapping frequency is fully applicable in the upper limb peripheral nerve injury detection, and the effects of rehabilitation treatment on upper limb peripheral nerve injury through finger tapping by comparing with before and after the treatment.

## Materials and methods

### Ethics statement

This study was approved by the Shanghai University of Sport Human Subjects Research Review Committee (Shanghai, China, Approval Number: 2014028, see Attachment 1). The study followed the protocols approved by the Ethics Committee, and the subjects' confidentiality was strictly maintained throughout the study.

### Participants

**Figure 1:**
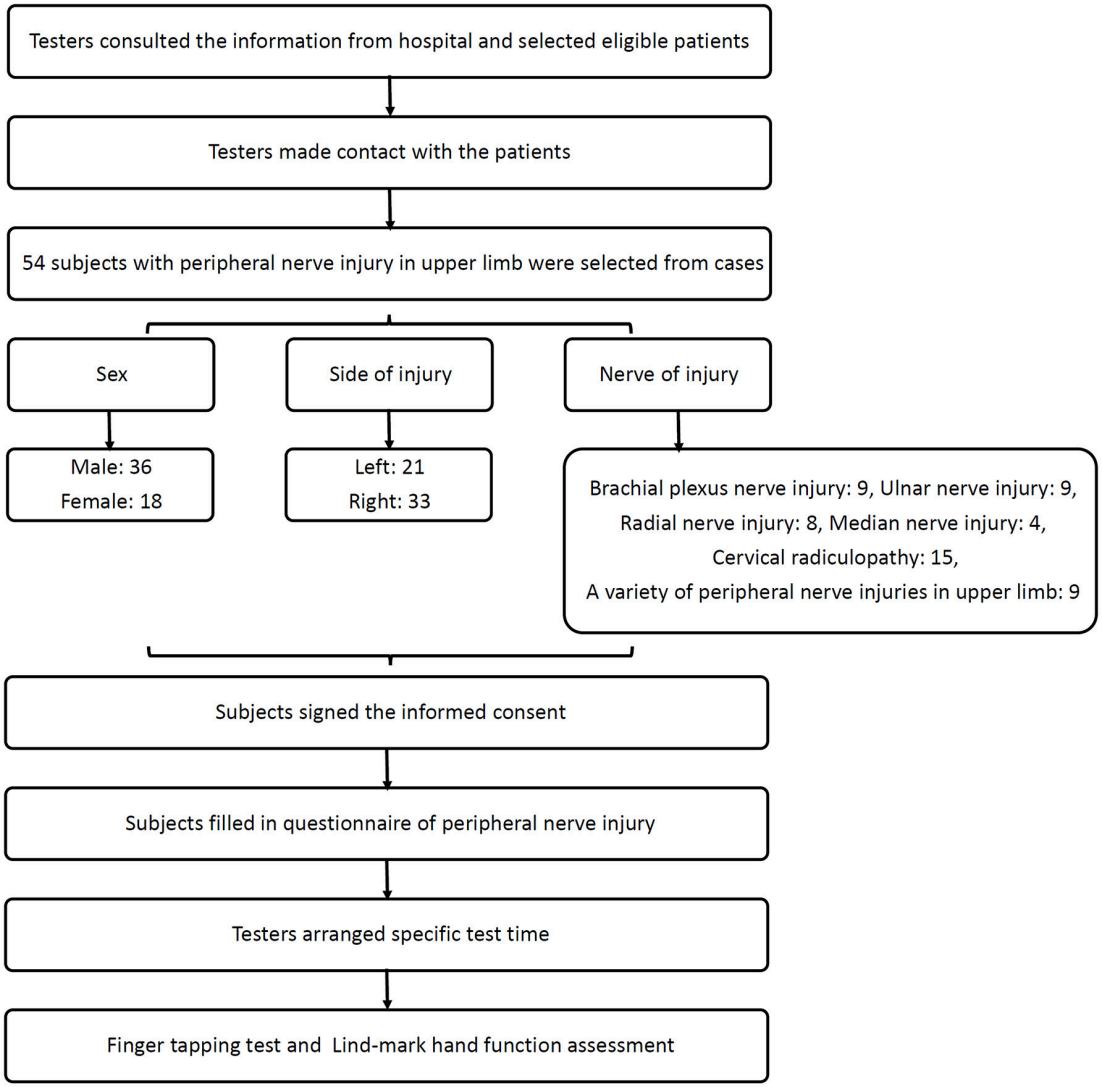
The flowchart of this study.

Here, 54 patients with upper limb peripheral nerve injury (36 males and 18 females) were selected from cases that were admitted to the Department of Hand Surgery and Rehabilitation Medicine, Huashan Hospital, Fudan University, Shanghai, China from July 2014 to April 2016. The average age of 36 males is 43.2 ± 14.9 years, and the average course of disease is 5.4 ± 4.3 months; the average age of 18 females is 49.7 ± 14.5 years, and the average course of disease is 4.3 ± 3.7 months. These cases included 21 subjects with left upper limb peripheral nerve injury and 33 subjects with right upper limb peripheral nerve injury. These cases covered 9 subjects with brachial plexus nerve injury, 9 with ulnar nerve injury, 8 with radial nerve injury, 4 with median nerve injury, 15 with cervical radiculopathy, and 9 with a variety of upper limb peripheral nerve injuries (Figure 1). By asking the patients and their family, we confirmed that all subjects were right-handed.

To what extent the nerve is injured was determined by medical history of the patient, examination was carried out by a nerve specialist as well as electrophysiological examination (Tao and Yang, 2009).

1. Examination on electromyography (EMG) and nerve conduction function was performed to check if the region of motor units consisted with the neuromuscular movement potential (Bendszus et al., 2004), serving as the gold standard for peripheral nerve function evaluation (Aagaard et al., 2003);
2. High-frequency ultrasonography was used to measure the location and damage of peripheral nerve;
3. MRI examination helped to make a distinction between the neuraxis rupture and nerve rupture based on difference in signal strength of short time inversion recovery (STIR), as well as characteristics of changes at different time points (Yu et al., 2010).

In addition to medical history, patients who met one of the above mentioned three criteria and had been determined by doctor to be patients with peripheral nerve injuries in upper limb were chosen as subjects for the present study.

Exclusion criteria:

1. Patients with central nerve injury;
2. Patients with no dysfunction of motor or sensory neurons, reflex action, among other functions, and in the upper body;
3. Patients with difficulty in listening comprehension and cooperation;
4. Patients who were left-handed before the disease.

### Finger tapping test

The examining instrument for finger tapping applied in the present study was granted by a patent (Yu et al., 2010), and the invention patent number is 200410017340.1 (Attachment 2). Finger tapping movements were detected with an infrared photoelectric sensor and related data were input into a computer through a serial port with a millisecond precision setting.

The index finger showed the fastest, most subtle and flexible reaction among all body parts. The data within the set time of 8 s were recorded. In a previous study, movement of the ring and little fingers was slower than that of the index and middle fingers in both the pianists and controls (Aoki et al., 2005). The subjects' age, gender, major in sports, and result comparisons were included in the software design to provide a reasonable proof for the test results (Zhang et al., 2014). In order to reduce the effect of fatigue on the speed of forefinger tapping, the test time was set to 8 s. Subjects were asked to sit with a normal posture during the test (head straight, eyes staring in front of the frame), metacarpophalangeal joint arch, the palm heel and three lateral fingers contacting the desktop, index finger extension stretch into the frame. Since there are individual differences in finger length, the swing angles differed to achieve the same oscillation amplitude. The height of the swing frame was fixed, so a finger tapping movement below the set height was excluded as invalid. The angle between the swing and the horizontal plane was limited upset to 30° to prevent excessive swing finger tapping movement.

Subjects started the formal test after 1–2 exercises. When the tester and the subject were both ready, the subject began to swing his/her index finger tap rapidly and repetitively as soon as he/she heard the starting gun and at the same time the instrument started timing automatically. The data were input into a computer within the 8 s set time.

### Lind-mark hand function assessment

Revised on the basis of Fugl-Meyer-Assessment (FMA) scale, the Lind-mark score set more details than FMA scale (Lindmark and Hamrin, 1987). Lind-mark hand function assessment includes (1) Flexion of five fingers; (2) Stretching of five fingers; (3) The tips of thumb and index finger meet together; (4) Grasping: grasp a stick with metacarpophalangeal joints straight and interphalangeal joints flexed; (5) Cross Grip: hold a piece of paper between thumb and index finger (thumb should be straight adducent); (6) Pinch grip: grip a pen with thumb and index finger; (7) Cylindrical grip: hold a cup with thumb and index finger with the part between them stretched; (8) Spherical grip: hold a tennis with five fingers apart. Each index includes the above eight movements with a maximum score of 24-point. Scoring criteria are shown in Table 1. Lind-mark score's testers ask subjects questions and give them a score based on their answers. Lind-mark hand function assessment score was only tested for the affected hand, as the value of Lind-mark hand function score for the unaffected hand was 24-point by default.

**Table 1:** Lind-mark hand function assessment criteria.

**Score**	**Criteria**
0	Gripping action cannot be completed
1	Gripping action can be completed with no ability to overcome slight resistance
2	Gripping an object up to 5 s with no ability to overcome medium resistance, uncoordinated or non-standard grasp
3	Gripping an object against larger resistance up to 5 s and releasing the hand like an ordinary person, normal grasp

### Design and procedure

The testers consulted with the doctors about the medical history of patients with nerve injury and chose eligible patients. They contacted with the subjects and their families, and explained the aim, procedure, and other points and also invited them to participate in this study. All subjects who agreed to participate signed the informed consent as well (Attachment 3). The testers read and explained the questionnaire to the subjects and helped them fill in the questionnaire (Attachment 4). The testers consulted with each patient to ensure that the patients could cooperate to complete the test actively and voluntarily. After 2-week rehabilitation treatment in Rehabilitation Medicine Department of Huashan Hospital affiliated to Fudan University (Shanghai, China), we conducted finger tapping test and Lind-mark hand function assessment on the 54 subjects again. There was no patient loss in the study.

### Statistical analysis

SPSS 13.0 software (Chicago, IL, USA) was applied to analyze the data, which were expressed as mean ± SD in Tables, and were expresses as mean ± SEM in Figures. The Pearson Correlation was used to analyze the association between finger tapping frequency and Lind-mark hand function assessment score. Statistical differences were calculated using paired-samples *T*-test. *P* values < 0.05 were considered statistically significant.

## Results

### Information of the subjects

By sex and the side of upper limb with peripheral nerve injury, we divided 54 patients with peripheral nerve injury in upper limb into 4 groups: G1 (male, peripheral nerve injury in the right upper limb), G2 (female, peripheral nerve injury in the right upper limb), G3 (male, peripheral nerve injury in the left upper limb), and G4 (female, peripheral nerve injury in the left upper limb). Table 2 shows the summarized baseline characteristics of the subjects for the present research project.

**Table 2:** Baseline characteristics of study participants of the present research project.

**Group**	**Sex**	**Side of injury**	**N**	**Age (year)**	**Course of disease (Month)**
G1	Male	Right	23	43.0 ± 15.2	4.9 ± 4.2
G2	Female	Right	10	50.6 ± 13.5	3.9 ± 3.5
G3	Male	Left	13	43.4 ± 14.9	6.3 ± 4.5
G4	Female	Left	8	48.6 ± 16.5	4.9 ± 4.1

### The correlation analysis of finger tapping frequency and lind-mark hand function assessment score

**Figure 2:**
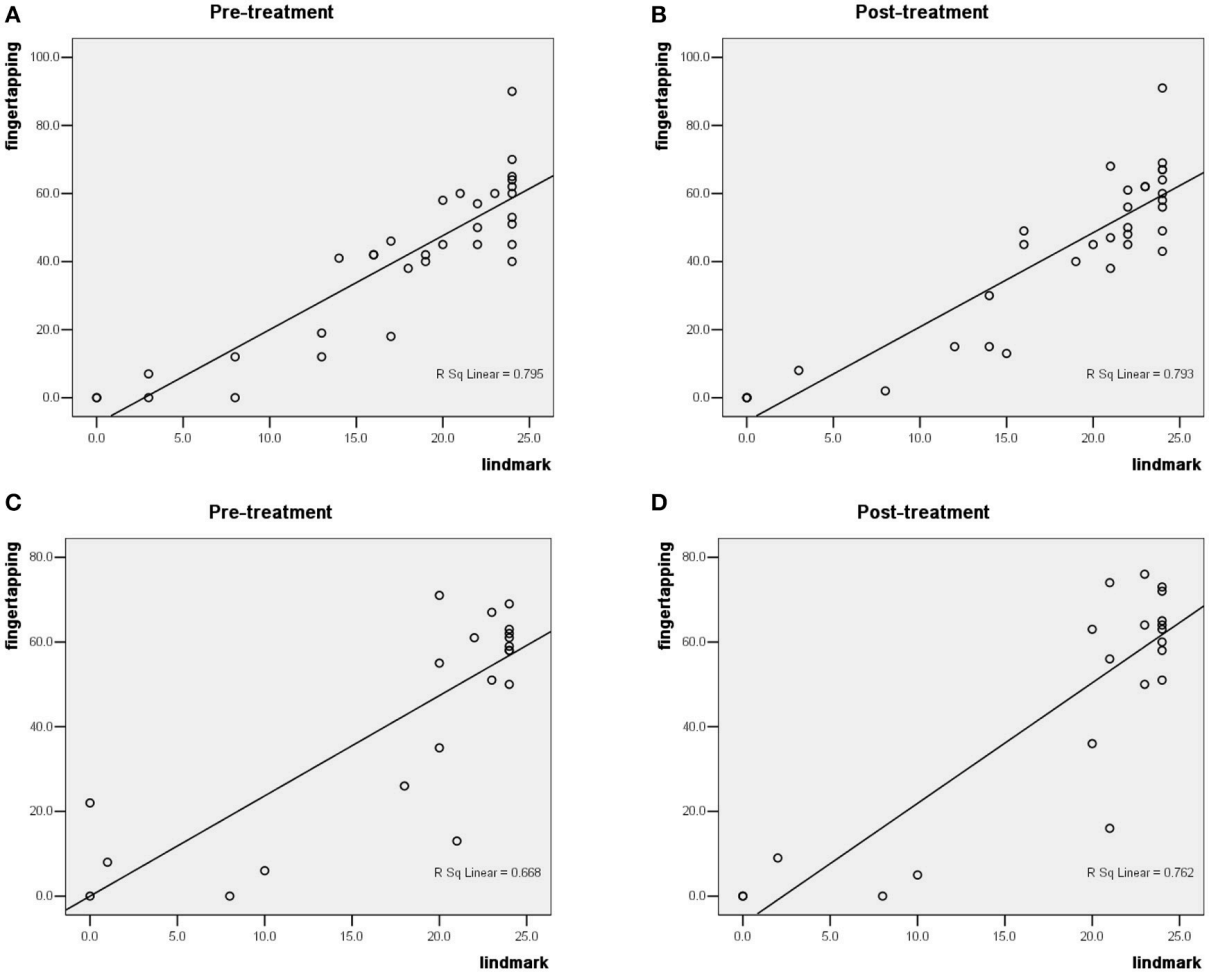
The finger tapping frequency and Lind-mark score of the 54 subjects. **(A,B)** show the correlation analysis of finger tapping frequency and Lind-mark score of 33 subjects with peripheral nerve injury in the right upper limb; **(C,D)** illustrate the correlation analysis of finger tapping frequency and Lind-mark score of 21 subjects with peripheral nerve injury in the left upper limb.

Figure 2A presents the correlation analysis of finger tapping frequency and Lind-mark hand function assessment score for 33 subjects in G1 and G2 with peripheral nerve injury in the right upper limb before treatment (*R*^2^ = 0.795,*P* = 0.000); Figure 2B shows that they have a high positive correlation after treatment (*R*^2^ = 0.793*, P* = 0.000). Figure 2C displays the correlation analysis of finger tapping and Lind-mark score of 21 subjects in G3 and G4 with peripheral nerve injury in the left upper limb before treatment (*R*^2^ = 0.668*, P* = 0.000); Figure 2D illustrates that they have a high positive correlation after treatment (*R*^2^ = 0.762, *P* = 0.000).

Table 3 compares the Lind-mark hand function assessment score and finger tapping frequency of the subjects before and after treatment in each group. We found that Lind-mark score did not change. Meanwhile, finger tapping frequency of the right affected hand was significantly higher after treatment than that of before treatment (male: *P* < 0.05; female: *P* < 0.01), suggesting that there is an effect of treatment, however, it is not the case with the left affected hand. We also found that finger tapping frequency of the unaffected hand of female subjects was significantly higher after treatment than that of before treatment (left: *P* < 0.01; right: *P* < 0.05), whereas the treatment had no similar effect on male subjects.

**Table 3:** Lind-mark score and finger tapping frequency comparison of pre-treatment and post-treatment.

**Group**			**G1**	**G2**	**G3**	**G4**
**Sex**			**Male**	**Female**	**Male**	**Female**
**Side of injury**			**Right**	**Right**	**Left**	**Left**
Lind-mark score	Left hand	Pre-treatment			16.9 ± 9.1	19.8 ± 7.9
		Post-treatment			17.1 ± 9.2	20.3 ± 7.5
	Right hand	Pre-treatment	15.6 ± 8.2	21.6 ± 3.0		
		Post-treatment	16.2 ± 8.5	22.3 ± 2.4		
Finger tapping frequency	Left hand	Pre-treatment	57.0 ± 19.1	61.7 ± 8.5	41.5 ± 28.4	44.5 ± 19.5
		Post-treatment	60.4 ± 15.9	66.1 ± 8.7**	41.5 ± 31.7	51.9 ± 20.6
	Right hand	Pre-treatment	34.8 ± 25.4	53.4 ± 9.1	65.2 ± 14.6	59.4 ± 16.3
		Post-treatment	37.5 ± 26.5*	56.0 ± 8.0**	67.2 ± 14.2	64.4 ± 16.9*

* *P < 0.05*,

** *P < 0.01, vs. pre-treatment*.

*Yellow means the unaffected hand of the subjects and pink means the affected hand of the patients*.

### Changes of parameters about different kinds of peripheral nerve injury in upper limb

Based on data analysis, we didn't find any correlation between finger tapping frequency and Lind-mark hand function assessment score of the affected hand with median nerve injury and ulnar nerve injury (data are not shown).

**Figure 3:**
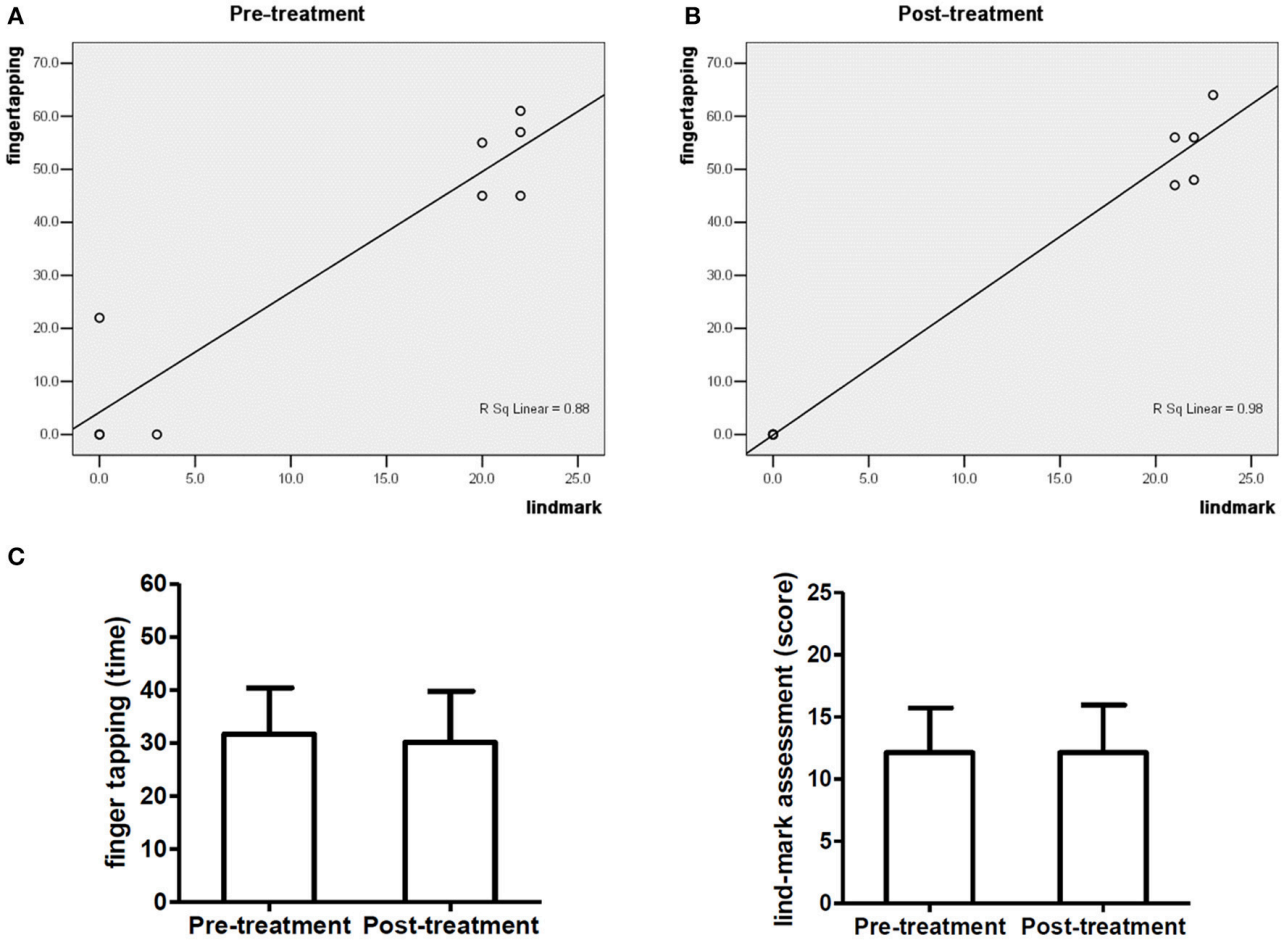
Baseline characteristics of 9 subjects with brachial plexus nerve injury. **(A,B)** show the correlation analysis of finger tapping frequency and Lind-mark score of 9 subjects with brachial plexus nerve injury; **(C)** compares the finger tapping frequency and Lind-mark score of 9 subjects.

Figure 3A shows the correlation analysis of finger tapping frequency and Lind-mark score of 9 subjects with brachial plexus nerve injury before treatment (*R*^2^ = 0.880, *P* = 0.000). Figure 3B demonstrates that finger tapping frequency and Lind-mark score of 9 subjects with brachial plexus nerve injury have a high positive correlation after treatment (*R*^2^ = 0.980, *P* = 0.000). Figure 3C illustrates there was no significant difference between finger tapping frequency and Lind-mark score of the affected hand of 9 subjects with brachial plexus nerve injury.

**Figure 4:**
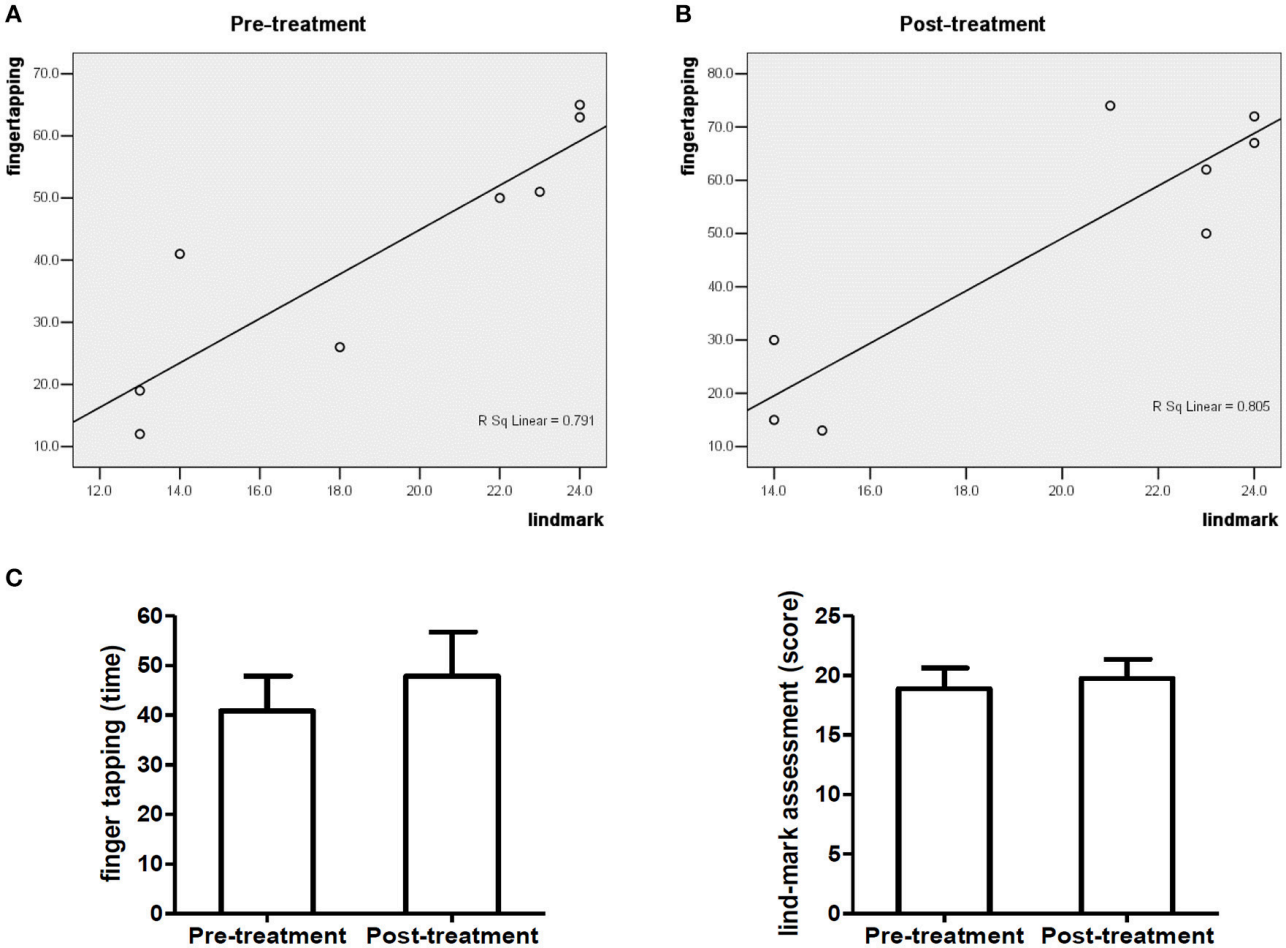
Baseline characteristics of 8 subjects with radial nerve injury. **(A,B)** show the correlation analysis of finger tapping frequency and Lind-mark score of 8 subjects with radial nerve injury; **(C)** compares the finger tapping frequency or Lind-mark score of 8 subjects with radial nerve injury.

The correlation analysis of finger tapping frequency and Lind-mark score of 8 subjects with radial nerve injury before treatment (*R*^2^ = 0.791*, P* = 0.003) is shown in Figures 4A,B presents that finger tapping frequency and Lind-mark score of 8 subjects with radial nerve injury have a high positive correlation after treatment (*R*^2^ = 0.805, *P* = 0.003). Figure 4C shows among 8 subjects with radial nerve injury, no significant difference was found between Lind-mark score and finger tapping frequency before and after treatment.

**Figure 5:**
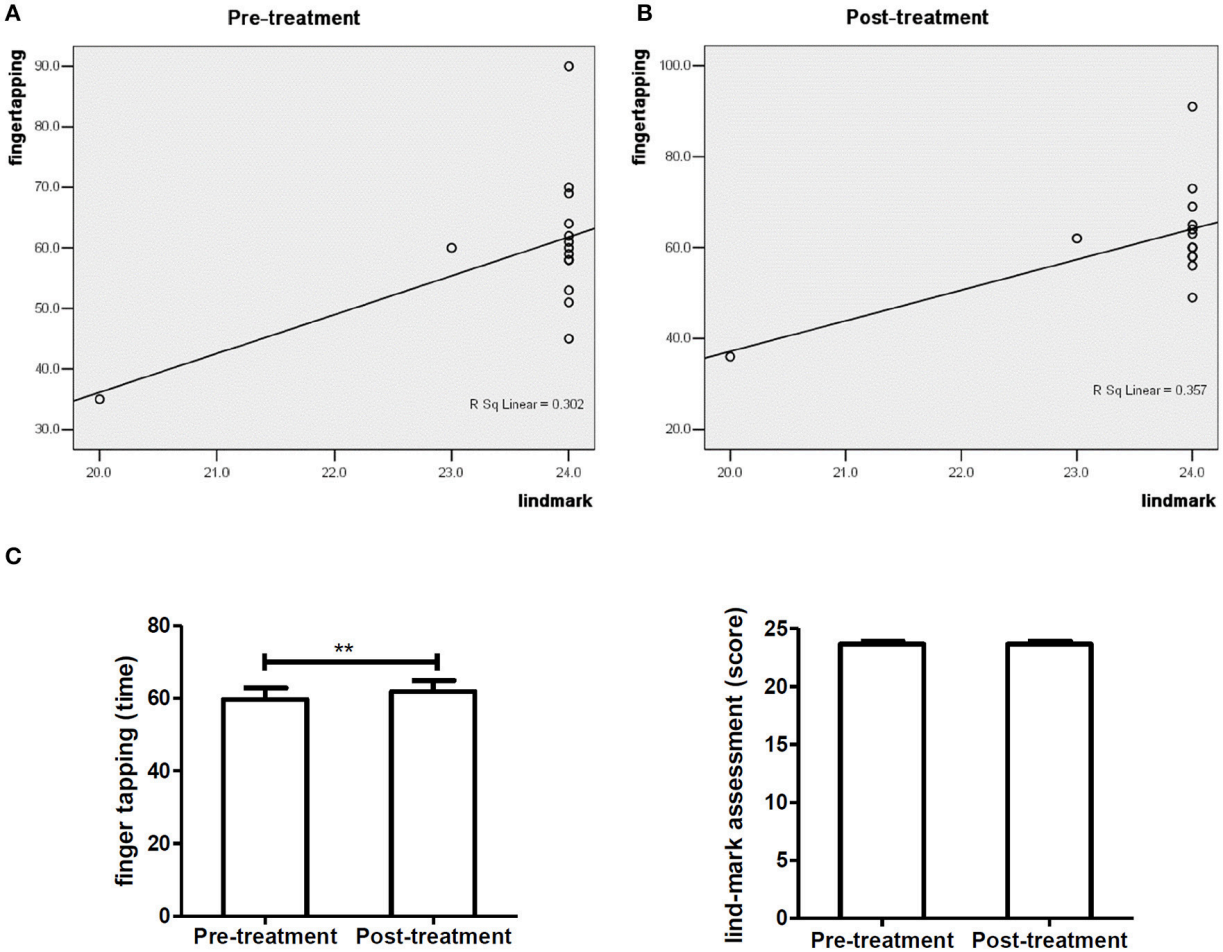
Baseline characteristics of 15 subjects with cervical radiculopathy. **(A,B)** show the correlation analysis of finger tapping frequency and Lind-mark score of 15 subjects with cervical radiculopathy; **(C)** compares the finger tapping frequency or Lind-mark score of 15 subjects with cervical radiculopathy.

Similarly, Figures 5A,B show that finger tapping frequency and Lind-mark score of 15 subjects with cervical radiculopathy have a low positive correlation (pre-treatment: *R*^2^ = 0.302, *P* = 0.034; post-treatment: *R*^2^ = 0.357, *P* = 0.019). Figure 5C shows among 15 patients with cervical radiculopathy, finger tapping frequency of the affected hand after treatment was significantly higher than that of before treatment (*P* < 0.01), however, no significant difference was observed Lind-mark score before and after treatment.

## Discussion

Repetitive rapid finger tapping is a common test of fine motor control of the upper extremities. Previous studies have proved that index finger tapping is significantly correlated with Lind-mark hand function assessment score and it plays an important role in hand function evaluation in patients with stroke (Zhang et al., 2014). The finger tapping is usually used to assess movement bradykinesia patients with Parkinson's disease patients (Lee et al., 2016). Moreover, a finger tapping test has been applied to measure performance validity in most of the standard neuropsychological evaluations (Axelrod et al., 2014). The computerized finger tapping test is highly efficient and precise in evaluating finger tapping speed as well as in the measurement of potential kinetic utility in research and clinical studies of motor performance (Hubel et al., 2013).

Normal finger tapping requires the functional integrity of the corticospinal tract, cerebellar motor circuitry, and proprioceptive pathways. Finger tapping is affected by many factors including hand dominance, age, gender, and neural control. All subjects in the present study were right-handed, and this was to ensure that the finger tapping test was not affected by differences in the dominant hand of subjects. We used paired-samples *T*-test to calculate statistical differences. The patients' data of finger tapping frequency and Lind-mark score before and after the rehabilitation treatment were compared. The comparison did not involve individual differences, therefore, there were no age differences. Sex differences were consistently found in tapping tasks with men who tapped faster than women (Roivainen, 2011; Hubel et al., 2013). Sex differences in the current study are associated with other factors that influence tapping frequency. To ensure statistical significance, the subjects were grouped by gender and the side of upper limb with peripheral nerve injury. Thus, we divided 54 patients with peripheral nerve injury in upper limb into four groups.

In a study conducted of (Aoki and Fukuoka, 2010), the subjects were asked to lie their hands flat with five fingers separate on the pressure sensor plane of the finger tapping testing apparatus during the test. In our research, we applied another frequently used finger swinging functional position, which is appropriate to ensure the index finger stretch up and down fast. This kind of hand gesture was similar to the hand gesture of subjects who grasped a mouse with their hands in a research performed by de Groot-Driessen et al. (2006). These two hand gestures were similar to each other, and they revealed that subjects swing their index finger in a good functional position. The hand functional position applied by Aoki and Fukuoka (2010) in the research was largely due to the special structure of the testing apparatus. In our research, before the formal testing, subjects were allowed to carry out 1-2 preliminary practices to become familiar with the testing instrument. A preliminary practice is commonly used in different researches, however, there was no report regarding the influence of preliminary practice on formal testing. It also deserves our consideration for the length of time of the trace effect of preliminary practice remains in human body.

On the whole, Figure 2 proves that finger tapping frequency and Lind-mark hand function assessment score have a high positive correlation regardless of the side of upper limb with peripheral nerve injury before and after the rehabilitation treatment. Therefore, finger tapping can be clinically used to detect changes of peripheral nerve injury in upper limb during rehabilitation therapy and subsequent recovery. A new system was designed to predict Lind-mark score based on features extracted from signals recorded during finger tapping. The Pearson's correlation coefficient for the finger tapping frequency and Lind-mark hand function assessment score of the right upper limb with peripheral nerve injury is relatively higher than the other side, which is likely related to right-handedness of the subjects. Hand dominance is an important factor affecting tapping rate (Hubel et al., 2013). In healthy individuals, tapping rate is ~10% faster in the dominant hand than non-dominant hand (Tanner and Bowles, 1995; Ashendorf et al., 2009), and with extended tapping there is less fatigue in the dominant than in the non-dominant hand (Hubel et al., 2013). No difference has been found in Lind-mark score of the affected hand before and after treatment. It should be pointed out that Lind-mark score of the unaffected hand is 24-point (as the maximum score), therefore, the testers didn't conduct Lind-mark hand function assessment on unaffected hand. However, whether the hand function of the unaffected hand of a patient with nerve injury is affected or improved after treatment has not been still clarified due to lack of accurate data to assess this problem. A particular instrument was used to measure the finger tapping frequency of the subjects to ensure high-accuracy of the data. Finger tapping frequency and Lind-mark score of the affected hand with peripheral nerve injury in upper limb have the same trend, and finger tapping frequency of the right affected hand after treatment was significantly higher than that of before treatment (male: *P* < 0.05; female: *P* < 0.01). It deserves attention that finger tapping frequency of the female subjects' unaffected hand after treatment was significantly higher than that of before treatment (left: *P* < 0.01; right: *P* < 0.05), however, this trend is different in male subjects after 2-week rehabilitation treatment. As men have a higher tapping rate than women in all age ranges (Hubel et al., 2013), it is more difficult to improve when the finger tapping frequency of unaffected hand has achieved a high point.

Neuroimaging studies suggest that the primary motor area of hand and the cerebellum plays a pivotal role in the control of finger tapping (Jäncke et al., 2004), while different kinds of peripheral nerve in upper limb contribute to this task as well. Direct repair and nerve autografting are primary options in the treatment of upper extremity with peripheral nerve injuries (Trehan et al., 2016), to date, the mechanism of injury, the course of disease, and the length of repair also play a key role on the rehabilitation treatment. We made the correlation analysis of finger tapping frequency and Lind-mark hand function assessment score of the subjects with different kinds of peripheral nerve injury in upper limb, and there was no a strong correlation between finger tapping frequency and Lind-mark score in the subjects with ulnar nerve injury and median nerve injury (data are not shown). Finger tapping frequency and Lind-mark score of the subjects with brachial plexus nerve injury and radial nerve injury have a high positive correlation. However, finger tapping frequency of the affected hand of patients with brachial plexus nerve injury or radial nerve injury did not change. Besides, Lind-mark score is the same between before and after treatment. Because the cases maybe covered subjects with brachial plexus nerve injury and radial nerve injury are less. Compared with Lind-mark hand function assessment, finger tapping frequency outperformed Lind-mark hand function assessment, because finger tapping frequency measures the functions of the affected hand as well as the index finger frequency of the unaffected hand of the patients. Moreover, the influence of testers on finger tapping data is lower than that of the Lind-mark hand function assessment score, as Lind-mark score's testers ask subjects questions and give them a score based on their answers. It is hard to avoid subjectivity. Finger tapping test records the times of the index finger tapping movement within 8 sec, which is slightly affected by the testers. Moreover, the influence of testers on data in conducting finger tapping test is less than the influence of testers on Lind-mark hand function assessment data. However, since finger tapping test is limited to the index finger, there will be difficulty in its elaboration to other kinds of nerve injury.

It is well-known that cervical radiculopathy is irrelevant to upper limb peripheral nerve, however, cervical radiculopathy often manifest itself as pain in one or both of the upper extremities or neck pain, which typically follows pressure or irritation on nerve roots in the cervical spine. It can be accompanied by motor, sensory, or reflex deficits and it often stems from degenerative disease in the cervical spine (Childress and Becker, 2016). Compared with Lind-mark score before treatment, it does not change after treatment, whereas finger tapping frequency of patients with cervical radiculopathy after treatment is significantly improved compared with that of before treatment.

## Author contributions

YuZ and LZ designed this study. LL, YiZ, RW, and YuZ performed experiments. LZ analyzed experimental data. LZ was responsible for manuscript writing. YuZ and XZ revised the manuscript. All authors approved the final version of this manuscript.

### Conflict of interest statement

The authors declare that the research was conducted in the absence of any commercial or financial relationships that could be construed as a potential conflict of interest.
